# In-depth characterization of neuroradiological findings in a large sample of individuals with autism spectrum disorder and controls

**DOI:** 10.1016/j.nicl.2022.103118

**Published:** 2022-07-16

**Authors:** Sara Ambrosino, Hasnaa Elbendary, Maarten Lequin, Dominique Rijkelijkhuizen, Tobias Banaschewski, Simon Baron-Cohen, Nico Bast, Sarah Baumeister, Jan Buitelaar, Tony Charman, Daisy Crawley, Flavio Dell'Acqua, Hannah Hayward, Rosemary Holt, Carolin Moessnang, Antonio M. Persico, Roberto Sacco, Antonia San José Cáceres, Julian Tillmann, the EU-AIMS LEAP Group, Eva Loth, Christine Ecker, Bob Oranje, Declan Murphy, Sarah Durston

**Affiliations:** a University Medical Center Utrecht, Utrecht, the Netherlands; b Clinical Genetics Department, Human Genetics and Genome Research Division of the National Research Center, Cairo, Egypt; c Department of Radiology, University Medical Center Utrecht, Utrecht, the Netherlands; f Department of Cognitive Neuroscience, Donders Institute for Brain, Cognition and Behaviour, Radboud University Nijmegen Medical Centre, Nijmegen, the Netherlands; d Department of Child and Adolescent Psychiatry, Central Institute of Mental Health, Medical Faculty Mannheim, University of Heidelberg, Mannheim, Germany; e Autism Research Centre, Department of Psychiatry, University of Cambridge, Cambridge, United Kingdom; g Department of Psychology, Institute of Psychiatry, Psychology & Neuroscience, King’s College London, London, United Kingdom; h Department of Forensic and Neurodevelopmental Sciences, Institute of Psychiatry, Psychology & Neuroscience, King’s College, London, United Kingdom; i Department of Psychiatry and Psychotherapy, Central Institute of Mental Health, University of Heidelberg, Mannheim, Germany; j Child and Adolescent Neuropsychiatry Program at Modena University Hospital, & Department of Biomedical, Metabolic and Neural Sciences, University of Modena and Reggio Emilia, Modena, Italy; k Child Neuropsychiatry / Neurodevelopmental Disorders Unit, University “Campus Bio-Medico”, Rome, Italy; n Instituto de Investigación Sanitaria Gregorio Marañón (IISGM), Madrid and CIBERSAM (Centro Investigación Biomédica en Red Salud Mental), Spain; o Roche Pharma Research and Early Development, Neuroscience and Rare Diseases, Roche Innovation Center Basel, F. Hoffmann–La Roche Ltd., Basel, Switzerland; l Department of Child and Adolescent Psychiatry, University Hospital, Goethe University, Frankfurt am Main, Germany; m Center for Neuropsychiatric Schizophrenia Research (CNSR) and Center for Clinical Intervention and Neuropsychiatric Schizophrenia Research (CINS), Mental Health Centre Glostrup, University of Copenhagen, Glostrup, Denmark

**Keywords:** Brain structural MRI, Radiological assessment, ASD

## Abstract

**Background:**

Autism spectrum disorder (ASD) is a group of neurodevelopmental conditions associated with quantitative differences in cortical and subcortical brain morphometry. Qualitative assessment of brain morphology provides complementary information on the possible underlying neurobiology. Studies of neuroradiological findings in ASD have rendered mixed results, and await robust replication in a sizable and independent sample.

**Methods:**

We systematically and comprehensively assessed neuroradiological findings in a large cohort of participants with ASD and age-matched controls (total N = 620, 348 ASD and 272 controls), including 70 participants with intellectual disability (47 ASD, 23 controls). We developed a comprehensive scoring system, augmented by standardized biometric measures.

**Results:**

There was a higher incidence of neuroradiological findings in individuals with ASD (89.4 %) compared to controls (83.8 %, *p* = .042). Certain findings were also more common in ASD, in particular opercular abnormalities (OR 1.9, 95 % CI 1.3–3.6) and mega cisterna magna (OR 2.4, 95 % CI 1.4–4.0) reached significance when using FDR, whereas increases in macrocephaly (OR 2.0, 95 % CI 1.2–3.2), cranial deformities (OR 2.4, 95 % CI: 1.0–5.8), calvarian / dural thickening (OR 1.5, 95 % CI 1.0–2.3), ventriculomegaly (OR 3.4, 95 % CI 1.3–9.2), and hypoplasia of the corpus callosum (OR 2.7, 95 % CI 1.1–6.3) did not survive this correction. Furthermore, neuroradiological findings were more likely to occur in isolation in controls, whereas they clustered more frequently in ASD. The incidence of neuroradiological findings was higher in individuals with mild intellectual disability (95.7 %), irrespective of ASD diagnosis.

**Conclusion:**

There was a subtly higher prevalence of neuroradiological findings in ASD, which did not appear to be specific to the condition. Individual findings or clusters of findings may point towards the neurodevelopmental mechanisms involved in individual cases. As such, clinical MRI assessments may be useful to guide further etiopathological (genetic) investigations, and are potentially valuable to fundamental ASD research.

## Highlights

• Neuroradiological findings are more common in autism spectrum disorder and tend to cluster.
• Neuroradiological findings are not specific to autism spectrum disorder.
• Neuroradiological findings may point toward developmental processes involved.
• Qualitative screening of brain MRI scans complements quantitative morphometry.

## 1 Introduction

Autism spectrum disorder (ASD) is a group of highly heterogeneous neurodevelopmental conditions and is related to differences in brain structure (Chen et al., 2011, Ecker et al., 2015, van Rooij et al., 2018). To date, structural neuroimaging studies in ASD have largely focused on *quantitative* data, with the most common findings being changes in brain morphometry in multiple brain regions (particularly in the striatal and fronto-temporal areas), combined with an atypical trajectory of brain growth in autistic individuals (Ecker et al., 2015, van Rooij et al., 2018). Specifically, enlarged brain volume in young children with ASD is one of the most consistent findings in autism research (Courchesne, 2002). The enlargement of the brain in ASD is accompanied by increased head circumference (Lainhart et al., 1997); it occurs during infancy and toddler years, while it is unclear whether it persists into later childhood and adolescence (Courchesne et al., 2001, Aylward et al., 2002). This suggests that the trajectory of early brain growth may be different in ASD (Courchesne et al., 2011). Later studies consistently found that early brain overgrowth in ASD differs between brain regions, mostly affecting frontal and temporal areas (Chen et al., 2011). Additionally, volumetric differences have been reported in numerous subcortical structures in ASD (Li et al., 2021), especially corpus callosum (Bellani et al., 2013, Frazier and Hardan, 2009), caudate nucleus (Qiu et al., 2016), and cerebellum (D'Mello et al., 2015), although these findings are less consistent. Discrepancies between quantitative neuroimaging studies in ASD may be due to methodological differences, and, notably, to the established wide phenotypic diversity, both clinical and neurobiological (neuroanatomical), among individuals in the autism group. Assessment of neuroanatomical variation at an individual rather than just at a group level may therefore be more suited to understanding individual differences in the neurobiological underpinnings of the autism spectrum.

*Qualitative* evaluation of individual scans is the typical approach in clinical neuroradiological practice. Several studies have investigated qualitative neuroradiological findings in ASD (Piven et al., 1990, Zeegers et al., 2006, Boddaert et al., 2009, Vasa et al., 2012, Erbetta et al., 2014, Erbetta et al., 2015, Monterrey et al., 2017, Myers et al., 2020). However, these reported inconsistent prevalence and characteristics of brain abnormalities in ASD, and as such are inconclusive. As a consequence, brain magnetic resonance imaging (MRI) is currently *not* included as a clinical standard when investigating autism (Filipek et al., 2000), although it may be indicated in cases with co-morbid conditions or neurological signs and symptoms (Schaefer et al., 2013, Cooper et al., 2016). Most qualitative studies used relatively small samples (N = 26 to 168) (Piven et al., 1990, Zeegers et al., 2006, Boddaert et al., 2009, Erbetta et al., 2014, Erbetta et al., 2015, Monterrey et al., 2017), and the methods were inconsistent between studies, with different inclusion criteria (e.g., with or without individuals with intellectual disability, ID), and different definitions of ‘neuroradiological findings’.

Abnormal findings on brain scans occur fairly commonly, in an estimated 20–25 % of the general population (Katzman et al., 1999, Kim et al., 2002, Jansen et al., 2017). However, they are highly heterogenous, and specific findings are infrequent, meaning that while the chances of finding any abnormality might be relatively large, the chances of finding a particular one are not. Studies of neuroradiological findings in ASD have used different categorization systems, and sometimes did deliberately not report certain findings, as they were considered not to be clinically relevant, or a variation of normal brain anatomy (Boddaert et al., 2009). Inevitably, these different approaches have contributed to the heterogenous nature of the findings reported in the literature. Notably, there is *no* agreed-upon definition of what constitutes a deviation from normal: brain structure demonstrates a wide variation in shape and size, and the range of normality is to some degree arbitrary, and, for some structures, simply unknown (Osborn and Preece, 2006).

Considering the intrinsic heterogeneity of the clinical and imaging data in ASD, and the relative infrequency of most reported neuroradiological findings as individual entities, studies using a comprehensive characterization of neuroradiological findings, and in large samples of participants, are essential. On a methodological level, it is important to screen brain scans explicitly for neuroradiological findings prior to quantitative analysis for a more comprehensive analytical and interpretative approach of brain morphology. Indeed, the presence of neuroanatomical defects often precludes certain imaging pipelines. Conventional neuroimaging studies in autism are therefore biased towards ‘typical’ brain anatomy. Furthermore, neuroradiological findings can point to developmental causal mechanisms (e.g., small cerebellum associated with pons hypoplasia, or with a posterior fossa cyst), and may therefore help elucidate the neurodevelopmental processes related to ASD.

Hence, this study aimed to systematically and comprehensively characterize qualitative brain MRI findings in a large sample of individuals with ASD, with and without ID, and matched controls. We capitalized on the large sample of the LEAP cohort (Charman et al., 2017, Loth et al., 2017), and constructed a comprehensive scoring system covering all brain structures and regions, permitting us to characterize in a standardized manner any visible morphological or signal abnormality identified on MRI scans including possible variants (neuroradiological findings), regardless of their clinical relevance. We expected to find an increased prevalence of structural brain abnormalities in individuals with ASD, especially in those with ID. Given the inconsistent literature, we were not able to derive specific hypotheses on which brain regions would be most affected.

## 2 Material and methods

### 2.1 Participants

We included participants of the Longitudinal European Autism Project (LEAP). The study design, methodologies, and clinical characterization of the LEAP cohort have been described extensively in previous publications (Charman et al., 2017, Loth et al., 2017). In short, participants were recruited and assessed across six research centres in Europe: Institute of Psychiatry, Psychology and Neuroscience, King’s College London (KCL, United Kingdom); Autism Research Centre, University of Cambridge (UCAM, United Kingdom); University Medical Centre Utrecht (UMCU, the Netherlands); Radboud University Nijmegen Medical Centre (RUNMC, the Netherlands); Central Institute of Mental Health (CIMH, Germany); and University Campus BioMedico (UCBM, Italy).

We included participants aged 6–30 years with intelligence quotient (IQ) in the typical range (75+), and participants with mild ID (IQ 50–74), aged 12–30 years. Females were purposely over-recruited in LEAP, with a targeted Male:Female ratio of 3:1, to enable better analysis of sex effects.

Inclusion criteria for the ASD sample were an existing clinical diagnosis of ASD according to the Diagnostic and Statistical Manual of Mental Disorders (DSM)-IV (American Psychiatric Association, 1994), DSM-IV-TR (American Psychiatric Association, 2000), DSM-5 (American Psychiatric Association, 2013) or ICD-10 (World Health Organization (WHO), 1993) criteria. In addition, the Autism Diagnostic Observation Schedule (ADOS, Lord et al., 2000, Lord et al., 2012) and the Autism Diagnostic Interview-Revised (ADI-R, Rutter et al., 2003) were administered to support the clinical diagnosis of ASD. However, individuals with a clinical diagnosis of ASD who did not reach cut-off on these instruments were not excluded, as clinical judgement was considered to be more stable and reliable than scores on individual diagnostic instruments per se (Charman and Gotham, 2013).

Participants were purposely recruited in LEAP to enable in depth experimental characterization of biological markers including the use of complex methodologies (e.g., MRI), and yet preserve the widest possible clinical diversity of the autism spectrum. Therefore, individuals with very low IQ (<50) were excluded in LEAP, while IQ ≥ 50 was included, and all psychiatric comorbidities were permitted, except for psychosis or bipolar disorder. Syndromic forms of intellectual disabilities were permitted. Participants on stable medication (minimum 8 weeks) at study entry and over the course of the study were included. Controls were excluded in case of any psychiatric morbidity. In all participants, additional exclusion criteria were uncorrectable hearing or visual impairments, any major neurological disorders, or the presence of metals in the body that precluded the MRI session (see Charman et al., 2017 for more details).

The study was approved by national and local independent ethics committees at each study site. Prior to testing, all participants, and/or their parents/legal guardian, provided written informed consent, as well as participants’ assent.

### 2.2 MRI data acquisition and quality assessment

Participants were assessed between January 2014 and March 2017 (LEAP wave 1). We acquired structural MRI scans from 704 participants, including high-resolution three-dimensional T1-weighted (T1w) scans (n = 695), T2-weighted (T2w) fast spin-echo scans (n = 411), and fluid attenuated inversion recovery (FLAIR) scans (n = 357). Scans were acquired on 3T scanners from different manufacturers (Siemens, General Electrics, Philips) across the six participating centers using the same acquisition protocol. The scanning parameters for the T1w, T2w, and FLAIR scans at each site are provided in Appendix A (Table A1).

All scans were assessed by a team of three raters based at the UMCU: one senior pediatric neuroradiologist (ML) with broad expertise in brain congenital anomalies, and two pediatric neurologists experienced in brain development and structural MRI assessment (SA, HE). Specifically, each scan was assessed by SA (rater 1), while the scans of 218/620 (35.2 %) participants were independently assessed by HE (rater 2). Additionally, the scans of 104/620 (16.8 %) participants were assessed by the neuroradiologist (ML). Agreements between the raters were between 92 and 98 % (see details below).

Scans were coded in order to ensure rater blindness to study site, participant identity and diagnosis at all times during analysis. However, raters were aware of the participants’ age at the time of scan to inform the interpretation of brain structure growth and maturation (i.e., myelination).

First, we evaluated the acquisition symmetry and overall quality of each scan using a 0 to 5 rating scale (5 for the best quality scan). In case of multiple acquisitions of the same sequence, the best quality-one was carried forward for analysis.

We excluded 80 participants for insufficient data quality (T1w quality < 3) due to missing T1w (n = 9), or to the presence of significant movement artifacts, primarily in the ASD group (n = 71; 58 ASD, 13 controls; *p* <.001). We additionally excluded 4 participants due to incomplete demographic information.

Eventually, we retained a final sample of 620 individuals (348 ASD, 272 controls), including 70 participants (11.3 %) with mild ID (47 ID-ASD, 23 ID-controls). The details of participant characteristics included in this study are provided in Table 1. The ASD and control groups were matched for age, but not for sex or IQ. The ASD group included more males (*p* =.048) and had lower full-scale IQ (difference 5.6 points; *p* <.001). The ID group included more males (*p* =.022) and had older participants (*p* =.002) compared to the group of participants with IQ in the typical range (details provided in Table 2). There were no between-groups differences in the distribution of the available T1w, T2w and FLAIR scans (*p* =.082).

**Table 1:** Demographic and clinical characteristics of the sample.

		ASD n = 348	Controls n = 272	Group differences*
Sex	n male - female	252–96	176–96	.048
Age at scan	Years M (SD)	17.6 (5.6)	17.5 (5.8)	.935
ID	n ID - typical IQ	47–301	23–249	.049
Total IQ	M (SD)	99.4 (19.0)	105.0 (18.0)	< .001
ADI-R	Social M (SD)	16.3 (6.9)	–	–
	Communication M (SD)	12.9 (5.7)	–	–
	RRB M (SD)	4.3 (2.7)	–	–
ADOS	Social M (SD)	5.9 (2.6)	–	–
	RRB M (SD)	4.7 (2.8)	–	–
	Total M (SD)	5.2 (2.7)	–	–
Scans per participant	n 1	89	91	.082
	n 2	115	86	
	n 3	144	95	
Scans in Total	n	751	548	–

Abbreviations: ASD, autism spectrum disorder; n, number; M, mean; SD, standard deviation; IQ, intelligence quotient; ID, intellectual disability; ADI-R, Autism Diagnostic Interview- Revised; RRB, restricted and repetitive behaviors; ADOS, Autism Diagnostic Observation Scale.

Note: *Χ^2^ for sex and number of scans acquired per person; *t* test for age and total IQ.

**Table 2:** Neuroradiological findings in the sample.

	Total N = 620	ASD n = 348	Controls n = 272	Group diff*	Typical IQ n = 550	ID n = 70	IQ diff*
Age (years M (SD))	17.6 (5.7)	17.6 (5.6)	17.5 (5.8)	ns	17.4 (5.8)	18.9 (4.4)	.002
Sex (n male – female)	428–192	252–96	176–96	.048	388–162	40–30	.022
**Head, brain, and lobes**							
Cranial deformity All	28 (4.5 %)	21 (6.0 %)	7 (2.6 %)	.039	23 (4.2 %)	5 (7.1 %)	ns
Plagiocephaly	9 (1.5 %)	6 (1.7 %)	3 (1.1 %)	ns	7 (1.3 %)	2 (2.9 %)	ns
Hyperbrachycephaly	9 (1.5 %)	8 (2.3 %)	1 (0.4 %)	ns	8 (1.5 %)	2 (2.9 %)	ns
Hyperdolichocephaly	10 (1.6 %)	7 (2.0 %)	3 (1.1 %)	ns	8 (1.5 %)	1 (1.4 %)	ns
Cranial volume All	133 (21.5 %)	87 (25.0 %)	46 (16.9 %)	.018	115 (20.9 %)	18 (25.7 %)	ns
Microcephaly	47 (7.6 %)	27 (7.8 %)	20 (7.4 %)	ns	36 6.5 %)	11 (15.7 %)	.007
Macrocephaly	86 (13.9 %)	60 (17.2 %)	26 (9.6 %)	.007	79 (14.4 %)	7 (10 %)	ns
Calvarian / dural thickening	126 (20.3 %)	81 (23.3 %)	45 (16.5 %)	.039	99 (18.0 %)	27 (38.6 %)	< .001
Opercular abnormality Bi/unilateral	229 (36.9 %)	150/0 (43.1 %)	77/2 (29.0 %)	< .001	191 (34.7 %)	38 (54.3 %)	.001
**Cerebral cortex**							
Malformations All	8 (1.3 %)	7 (2.0 %)	1 (0.4 %)	ns	5 (0.9 %)	3 (4.3 %)	ns
Periventricular nodular Heterotopia	3 (0.5 %)	2 (0.6 %)	1 (0.4 %)	ns	2 (0.4 %)	1 (1.4 %)	ns
Simplified gyral pattern	2 (0.3 %)	2 (0.6 %)	0 (0 %)	ns	2 (0.4 %)	0 (0 %)	ns
Cortical dysplasia	3 (0.5 %)	3 (0.9 %)	0 (0 %)	ns	1 (0.2 %)	2 (2.9 %)	ns
Lesion	3 (0.5 %)	2 (0.6 %)	1 (0.4 %)	ns	2 (0.4 %)	1 (1.4 %)	ns
**Hippocampi**							
Lesion	2 (0.3 %)	1 (0.3 %)	1 (0.4 %)	ns	2 (0.4 %)	0 (0 %)	ns
**White matter**							
Lesion	15 (2.4 %)	10 (2.9 %)	5 (1.8 %)	ns	12 (2.2 %)	3 (4.3 %)	ns
**Virchow-Robin spaces**							
Dilation All	338 (54.5 %)	189 (54.3 %)	149 (54.8 %)	ns	302 (54.9 %)	36 (51.4 %)	ns
Deep white matter / subcortical	133 (21.5 %)	66 (19.0 %)	67 (24.6 %)	ns	120 (21.8 %)	13 (18.6 %)	ns
Lenticulo-striate	300 (48.4 %)	171 (49.1 %)	129 (47.4 %)	ns	268 (48.7 %)	32 (45.7 %)	ns
**Basal ganglia**	0 (0 %)	0 (0 %)	0 (0 %)	ns	0 (0 %)	0 (0 %)	ns
**Posterior fossa**							
All	115 (18.5 %)	80 (23.0 %)	35 (12.9 %)	.002	96 (17.5 %)	19 (27.1 %)	ns
Dandy-Walker complex	106 (17.1 %)	73 (21.0 %)	33 (12.1 %)	.004	90 (16.4 %)	16 (22.9 %)	ns
Mega-cisterna magna	82 (13.2 %)	60 (17.2 %)	22 (8.1 %)	.001	76 (13.8 %)	6 (8.6 %)	ns
Dandy-Walker variant	3 (0.5 %)	1 (0.3 %)	2 (0.7 %)	ns	0 (0 %)	3 (4.3 %)	ns
Blake pouch cyst	1 (0.2 %)	1 (0.3 %)	0 (0 %)	ns	1(0.2 %)	0 (0 %)	ns
Arachnoid cyst	13 (2.1 %)	6 (1.7 %)	7 (2.6 %)	ns	12 (2.2 %)	1 (1.4 %)	ns
Vermian hypoplasia	7 (1.1 %)	5 (1.4 %)	2 (0.7 %)	ns	1 (0.2 %)	6 (8.6 %)	< .001
Chiari type 1 malformation	7 (1.1 %)	5 (1.4 %)	2 (0.7 %)	ns	5 (0.9 %)	2 (2.9 %)	ns
Lesion	1 (0.2 %)	1 (0.3 %)	0 (0 %)	ns	1 (0.2 %)	0 (0 %)	ns
Vascular anomaly	1 (0.2 %)	1 (0.3 %)	0 (0 %)	ns	0 (0 %)	1 (1.4 %)	ns
**CSF spaces**							
Ventriculomegaly	26 (4.2 %)	21 (6.0 %)	5 (1.8 %)	.010	22 (4.0 %)	4 (5.7 %)	ns
Cavum septum pellucidum / vergae	12 (1.9 %)	4 (1.1 %)	8 (2.9 %)	ns	10 (1.8 %)	2 (2.9 %)	ns
Choroid plexus cysts	3 (0.5 %)	2 (0.6 %)	1 (0.4 %)	ns	1 (0.2 %)	2 (2.9 %)	ns
Subarachnoid spaces Enlargement	57 (9.2 %)	34 (9.8 %)	23 (2.9 %)	ns	60 (10.9 %)	19 (27.1 %)	< .001
Calcifications	2 (0.3 %)	0 (0 %)	2 (0.7 %)	ns	2 (0.4 %)	0 (0 %)	ns
**Midline**							
CC Hypoplasia All	30 (4.8 %)	23 (6.6 %)	7 (2.6 %)	.020	17 (3.1 %)	13 (18.6 %)	< .001
CC thin	10 (1.6 %)	7 (2.0 %)	3 (1.1 %)	ns	5 (0.9 %)	5 (7.1 %)	< .001
CC short	12 (1.9 %)	8 (2.3 %)	4 (1.5 %)	ns	7 (1.3 %)	5 (7.1 %)	.001
CC short and thin	4 (0.6 %)	4 (1 %)	0 (0 %)	ns	2 (0.4 %)	2 (2.9 %)	ns
CC partial agenesis	1 (0.2 %)	1 (0.3 %)	0 (0 %)	ns	0 (0 %)	1 (1.4 %)	ns
CC focal hypoplasia	4 (0.6 %)	4 (1.1 %)	0 (0 %)	ns	3 (0.5 %)	1 (1.4 %)	ns
Pineal gland cyst All	90 (14.5 %)	54 (15.5 %)	36 (13.2 %)	ns	76 (13.8 %)	14 (20 %)	ns
≥ 10 mm	14 (2.3 %)	8 (2.3 %)	6 (2.2 %)	ns	9 (1.6 %)	5 (7.1 %)	.015
< 10 mm	76 (12.3 %)	46 (13.2 %)	30 (11.0 %)	ns	67 (12.2 %)	9 (12.9 %)	ns
**Other**							
Vascular anomalies All	10 (1.6 %)	7 (2.0 %)	3 (1.1 %)	ns	10 (1.8 %)	2 (2.9 %)	ns
DVA	8 (1.3 %)	6 (1.7 %)	2 (0.7 %)	ns	6 (1.1 %)	2 (2.9 %)	ns
Kissing carotids	1 (0.2 %)	0 (0 %)	1 (0.4 %)	ns	1 (0.2 %)	0 (0 %)	ns
Capillary teleangectasia	1 (0.2 %)	1 (0.3 %)	0 (0 %)	ns	1 (0.2 %)	0 (0 %)	ns
Cysts All	8 (1.3 %)	5 (1.4 %)	3 (1.1 %)	ns	7 (1.3 %)	1 (1.4 %)	ns
Arachnoid**	3 (0.5 %)	2 (0.6 %)	1 (0.4 %)	ns	3 (0.5 %)	0 (0 %)	ns
Poroencefalic	3 (0.5 %)	2 (0.6 %)	1 (0.4 %)	ns	2 (0.4 %)	1 (1.4 %)	ns
Neuroglial	1 (0.2 %)	0 (0 %)	1 (0.4 %)	ns	1 (0.2 %)	0 (0 %)	ns
Inclusion	1 (0.2 %)	1 (0.3 %)	0 (0 %)	ns	1 (0.2 %)	0 (0 %)	ns

Abbreviations: ASD, autism spectrum disorder; N or n, number; M, mean; SD, standard deviation; ns, not significant; IQ, intelligence quotient; ID, intellectual disability; CSF, cerebral spinal fluid; CC, corpus callosum; DVA, developmental venous anomalies.

Note: *Χ^2^ or Fisher’s Exact Test, as appropriate, for testing for groups differences on ASD and ID (raw p-values; results reaching significance when controlling FDR are indicated in bold).

**Arachnoid cysts in locations other than the posterior fossa.

In the final, high-quality sample, T1-weighted images were available for 100 % of the participants (620 scans); T2-weighted and FLAIR scans were available for 369 (59.5 %) and 310 (50 %) participants respectively, resulting in a total of 1299 scans. The overall visual quality of the included scans was 4.3/5, and did not differ between the ASD and control group (*p* =.082).

### 2.3 Brain MRI assessment

Special attention was paid to abnormalities previously described in the literature on neuroradiological findings in ASD (Piven et al., 1990, Taber et al., 2004, Zeegers et al., 2006, Boddaert et al., 2009, Vasa et al., 2012, Erbetta et al., 2014), or in related neuropsychiatric disorders (Nopoulos et al., 2000, Vasa et al., 2012, Sommer et al., 2013), and in healthy adult and pediatric populations (Katzman et al., 1999, Kim et al., 2002, Jansen et al., 2017). But primarily, our aim was to capture all potentially relevant neuroradiological findings. Arguably, some findings may not be clinically relevant to ASD, yet they may still be scientifically relevant, as they may point to neurodevelopmental mechanisms and provide information on the biological pathways involved. Therefore, we constructed a systematic and comprehensive scoring system (see Appendix B), covering all brain structures and regions, and characterizing all visibly detectable neuroradiological abnormalities (brain lesions, malformations, and anatomical variants). To enable quantification, we categorized our extensive assessment data into ten categories as follows: anomalies of 1) skull, whole brain, and brain lobes, 2) cerebral cortex, 3) hippocampi, 4) white matter, 5) Virchow-Robin (VR) perivascular spaces, 6) basal ganglia, 7) posterior fossa, 8) cerebral spinal fluid spaces, 9) midline, and 10) other.

Further, we compared the assessments performed by any two raters in each category (on 276 participants, for a total of 2760 observations) using a binary system. Readings were rated as ‘0′ in case of congruent descriptions (both readers rated the category as normal, or described the same type of abnormality), or ‘1′ in case of substantial differences. The agreement between rater 1 and 2, performed on 172 participants, was 92 %; the agreement between rater 1 and rater 3 (an experienced neuroradiologist), performed on 57 participants, was 98 %. In case of disagreement (a rating of ‘1′), consensus was reached by discussion.

Finally, we acquired multiple biometric measures from the MR-scans, using standardized methods and age-sex normed values. This permitted us to further characterize specific brain features and to objectively quantify anomalies, while accounting for sex and age effects.

Specifically, we acquired metrics of the whole head shape and size (Allanson et al., 2009, Franco et al., 2013), of the anterior and posterior inter-opercular distances (Chen et al., 1995, Chen et al., 1996), of the corpus callosum (length and thickness) (Garel et al., 2011, Karakaş et al., 2011), and of the lateral ventricles (Sarı et al., 2015). Further, we measured perivascular VR spaces (Heier et al., 1989), pineal gland cysts, and the length of the cavum septum pellucidum and cavum vergae (Dremmen et al., 2019) when present. In the posterior fossa, we measured the width of cisterna magna (Limperopoulos et al., 2008), and the extent of tonsillar ectopia in case of Chiari malformation type 1 (Baisden, 2012).

All measures were acquired on T1w sequences using the submillimeter caliper of MedINRIA medical image visualization software (https://med.inria.fr). For further details on the measurement’s methods and standard references, we refer to Appendix C. Notably, this data is distinct and provides complementary information to measures obtained using standard automated imaging pipelines such as FreeSurfer (Fischl, 2012). In fact, some of these measures, such as the distinct dimensions of the corpus callosum, may be related to different neurodevelopmental mechanisms (De León Reyes et al., 2020).

### 2.4 Statistical analysis

We conducted all statistical analyses using SPSS statistical package v26. We used Chi-squared or Fisher’s Exact Test, as appropriate, to analyze differences in the observed MRI findings between diagnostic groups (ASD vs controls), and between individuals with and without ID. Odds ratios [OR] were calculated to estimate the strength of the association between neuroradiological findings and ASD or ID. We applied a false discovery rate (FDR) correction (Benjamini and Hochberg, 1995) to control for multiple comparisons, using a significance threshold of *p* <.05. Notably, previous studies on neuroradiological findings in ASD have reported uncorrected results (e.g., Erbetta et al., 2014) due to their small number of comparisons. Hence, to enable better comparisons with previous studies, and in consideration of our novel, more rigorous assessment of the scans, we report both FDR-corrected and uncorrected results here.

Further, we explored the possibility of clustering. We compared the number of neuroradiological findings per individual (clusters) between groups. Then, we investigated the distribution of each type of finding within the clusters using Chi-square goodness of fit test. Post-hoc analyses were performed using adjusted standardized residuals for chi-square tests (Beasley and Schumacker, 1995). Finally, we performed pairwise correlations of the frequency distributions of neuroradiological findings in the sample, Bonferroni-corrected for multiple comparisons, and compared these correlations between groups.

## 3 Results

**Fig. 1:**
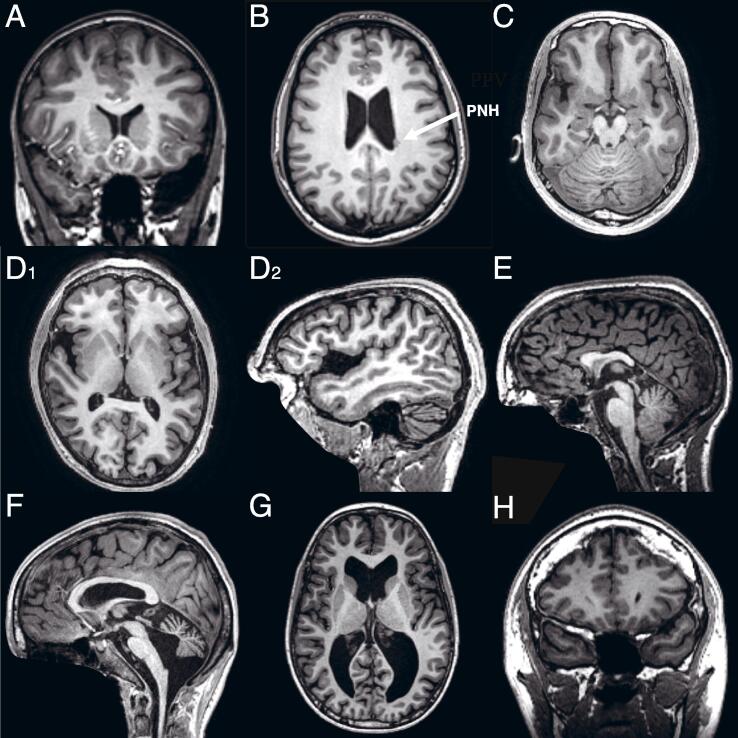
Examples of neuroradiological findings. Panel A: arachnoid cyst in the left temporal pole; Panel B: periventricular nodular heterotopia (PNH, arrow); Panel C: simplified gyral pattern; Panel D_1-2_: opercular abnormality; Panel E: partial agenesis of the corpus callosum; Panel F: Dandy-Walker variant; Panel G: ventriculomegaly; Panel H: thickening of the dura mater.

Complete demographic and high-quality brain MRI scans were available from 620 participants, aged from 6.8 to 30.6 years (Table 1). Table 2 summarizes the observed neuroradiological findings and between-group differences. Examples of common findings, and of some rarer anomalies encountered in our dataset, are depicted in Fig. 1.

### 3.1 Types of neuroradiological findings

Most participants (539/620, 86.9 %) had at least one neuroradiological finding, including 331/348 (89.4 %) participants with ASD, 95.7 % of participants with mild ID (44/47 ID-ASD, 23/23 ID-controls), and 205/249 (82.3 %) typically developing controls. Participants with ASD, and participants with mild ID irrespective of ASD diagnosis, were more likely to have a neuroradiological finding compared to their respective controls (*Χ^2^_1_* = 4.1*, p* =.042 and *Χ^2^_1_* = 5.3*, p* =.021, respectively). There was no difference in the frequency of total findings between males and females. Preliminary analysis on sex differences is provided in Appendix D. Among a few other results, we found a higher incidence of mega cisterna magna in males (*Χ^2^_1_* = 17.7; *p* <.001*, FDRp* <.001; OR 4.2, 95 % CI 2.0–8.5) (Table D1).

In ASD, we found a higher incidence of opercular abnormalities (*Χ^2^_1_* = 12.9*, p* <.001, FDR-adjusted value of *p (FDRp)* = 0.017; OR 1.9, 95 % CI 1.3–2.6), and mega cisterna magna (*Χ^2^_1_* = 11.1*, p* =.001, *FDRp* =.026; OR 2.4, 95 % CI 1.4–3.9), compared to controls (Table 2). Given the sex mismatch between diagnostic groups in our research population (Table 1), and the sex difference on mega cisterna magna (Table D1), we repeated the ASD analysis on mega cisterna magna in a male only subgroup (n = 428), which yielded consistent results (*p* =.009).

We additionally found a higher incidence in ASD of cranial deformities (*Χ^2^_1_* = 4.2*,* p =.039; OR 2.4, 95 % CI 1.0–5.8), macrocephaly (*Χ^2^_1_* = 7.5*, p* =.006; OR 2.0, 95 % CI 1.2–3.2), calvarian / dural thickening (*Χ^2^_1_* = 4.3*, p* =.039; OR 1.5, 95 % CI 1.0–2.3), ventriculomegaly (enlarged ventricles; (*Χ^2^_1_* = 6.7*, p* =.010; OR 3.4, 95 % CI 1.3–9.2), and hypoplasia of the corpus callosum (*Χ^2^_1_* = 5.4*, p* =.020; OR 2.7, 95 % CI 1.1–6.3), although these results failed to reach significance after correction for multiple comparison (Table 2). All these differences did not hold in the subsample of participants with mild ID (i.e. when comparing ID-ASD to ID-controls).

Instead, participants with mild ID had a higher incidence of microcephaly (*Χ^2^_1_* = 7.5*, p* =.006, *FDRp* =.042; OR 2.7, 95 % CI 1.3–5.5), calvarian / dural thickening (*Χ^2^_1_* = 16.2*, p* <.001, *FDRp* =.001; OR 2.9, 95 % CI 1.7–4.9), opercular abnormalities (*Χ^2^_1_* = 10.2*, p* =.001, *FDRp* =.011; OR 2.2, 95 % CI 1.4–3.7), vermian hypoplasia (*Χ^2^_1_* = 39.2*, p* <.001, *FDRp* <.001; OR 51.5, 95 % CI 6.1–434.3), enlargement of the subarachnoid spaces (*Χ^2^_1_* = 11.0*, p* =.001, *FDRp* =.008; OR 3.0, 95 % CI 1.5–5.7), and hypoplasia of the corpus callosum (*Χ^2^_1_* = 32.3*, p* <.001, *FDRp* =.002; OR 7.2, 95 % CI 3.3–15.5) compared to participants with IQ in the typical range (ASD and controls combined), see Table 2.

### 3.2 Clustering of neuroradiological findings

The number of neuroradiological findings per individual ranged from 0 to 8, regardless of clinical diagnosis. In more than half of cases, any single neuroradiological finding was accompanied by others: of the 620 participants, 81 (13.1 %) had no findings at all (‘cluster 0′), 189 (30.5 %) had one finding (‘cluster 1′), 142 (22.9 %) had two findings (‘cluster 2′), 119 (19.2 %) had three findings (‘cluster 3′), and 89 (14.4 %) had four or more findings (‘cluster 4+’).

**Fig. 2:**
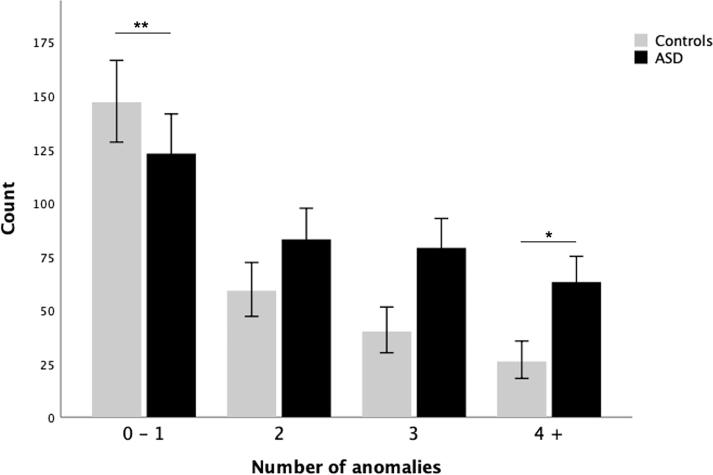
Clustering of neuroradiological findings in ASD vs controls. The number of neuroradiological findings per person differed between ASD and controls (p <.001). Asterisks mark significant group differences from post-hoc analysis: individuals with 0–1 neuroradiological findings were more prevalent in the control group (**p <.001), individuals with 2–3 findings did not differ between groups, individuals with 4 or more findings were significantly more prevalent in ASD compared to controls (*p =.029).

The number of neuroradiological findings per person differed between ASD and controls (*Χ^2^_3_* = 25.4*, p* <.001). Post-hoc analysis of the adjusted standardized residuals showed that individuals with 0 or 1 findings were more prevalent in the control group (*p* <.001), the number of individuals with 2–3 neuroradiological findings did not differ between groups, while individuals with 4 or more neuroradiological findings were more prevalent in the ASD group than in controls (*p* =.029), see Fig. 2. These differences were not present in the subsample of participants with mild ID.

Similarly, the number of neuroradiological findings per person differed between individuals with mild ID and individuals with typical IQ, irrespective of ASD diagnosis (*Χ^2^_3_* = 25.0*, p* <.001). Post-hoc analysis showed that individuals with 0 or 1 finding were more prevalent in the control group (*p* =.001), the number of individuals with 2–3 neuroradiological findings did not differ between the groups, and individuals with 4+ neuroradiological findings were more prevalent in mild ID than in participants with typical IQ (*p* =.001). These group differences were not present in the subsample of individuals with ASD (i.e. when comparing ID-ASD vs ASD with typical IQ).

### 3.3 Types by number of neuroradiological findings

We used Chi-square goodness of fit test and post-hoc analyses to explore if there was a difference in the frequency of different types of neuroradiological findings across the (1 to 4+) clusters. We found that macrocephaly, the mega cisterna magna, vermian hypoplasia, enlargement of the subarachnoid spaces and of lateral ventricles were more prevalent in the 4+ cluster (all *p_s_* < .001). In other words, these findings were commonly accompanied by three or more other findings. There were no differences between diagnostic groups in the distribution of any neuroradiological finding within the (1 to 4+) clusters, with the exception of the Virchow-Robin (VR) perivascular spaces: post-hoc analyses showed that the VR spaces were more prevalent (*p* =.001) in cluster 1 in controls (i.e., they occurred in isolation), whereas they were more prevalent in cluster 4+ (*p* =.012) in ASD.

Next, we investigated the correlation between each type of neuroradiological finding in the whole group. Due to the large number of comparisons, here we applied Bonferroni correction (adjusted *p* value = .0002). Results are summarized in Appendix D (Table D2). We found that macrocephaly was associated with calvarian / dural thickening and with ventriculomegaly, and that the microcephaly was associated with hypoplasia of the corpus callosum. We also found significant correlations between cortical malformations and cystic or WM abnormalities (all *p_s_* < .0001).

Finally, we tested whether correlation coefficients differed between the groups. We found no differences in these correlations in ASD, nor in individuals with mild ID, compared to controls.

## 4 Discussion

We performed a large study (N = 620) investigating neuroradiological findings in ASD. We used a systematic and comprehensive scoring system of brain findings on MRI, augmented with standardized biometric measures of brain features. We found that neuroradiological findings were more common and clustered more frequently in ASD, although this did not appear to be specific to the condition.

The incidence of brain MRI findings in ASD in our study was higher (89.4 %) than in previous reports, which ranged from 11 % (Vasa et al., 2012), 40–54 % (Piven et al., 1990, Taber et al., 2004, Zeegers et al., 2006, Boddaert et al., 2009: Erbetta et al., 2014, Erbetta et al., 2015, Myers et al., 2020), to 68 % (Monterrey et al., 2017) in ASD. This is likely related to several factors, such as the inclusion of participants with ID in our study (as there were more findings in ID-participants compared to participants with typical IQ), and our acquisition of extensive assessment data, which permitted us to report on a wide range of anatomical features, from common to more rare variants in brain anatomy.

The presence of specific neuroradiological markers may hint at the neurodevelopmental processes involved. For example, we found a higher incidence of mega cisterna magna in ASD, consistent with previous neuroradiologic studies (Erbetta et al., 2014, Erbetta et al., 2015). Mega cisterna magna is a cystic malformation of the posterior fossa characterized by a focal enlargement of the subarachnoid space located below the cerebellum (cerebellomedullary cistern), with normal fourth ventricle and cerebellar structures (Whitney et al., 2013, Bosemani et al., 2015). Mega cisterna magna is considered to be on the mild end of the Dandy-Walker (DW) complex, a wide spectrum of congenital abnormalities sharing overlapping radiological features, a similar clinical spectrum, and related developmental origins (Barkovich et al., 1989). Embryologically, the DW complex is thought to be related to insults predominantly involving the developing cerebellar hemispheres (leading to the DW variant, Fig. 1 panel F), or the developing fourth ventricle (associated to the mega cisterna magna), or both (leading to the most severe DW malformation) (Barkovich et al., 1989). Interestingly, in our study, we found a higher incidence of the DW complex as a whole in the autism group, largely driven by the presence of mega cisterna magna. This implicates the developmental period when the fourth ventricle develops, up to 26 weeks’ gestation (Brocklehurst, 1969), in ASD.

Prior studies on cerebellar morphometry provided variable results on the direction and pattern of differences observed in ASD (Brambilla et al., 2003, Donovan and Basson, 2017). Our study reflects such variability as well, as by definition the DW variant and malformation are characterized by varying degrees of malformation of the cerebellar regions, whereas in mega cisterna magna these structures are visually intact (Barkovich et al., 1989). The qualitative assessment of posterior fossa as conducted in our study may be able to help to stratify ASD into subtypes based on cerebellar anomalies at a macroscopic level.

Consistent with previous volumetric studies of the corpus callosum (CC) in ASD (reviewed by Bellani et al., 2013), we found a higher incidence of hypoplasia of the CC in the autism group, although this result did not reach significance when controlling FDR. The CC is the largest commissural white matter tract, involved in the integration of high-order functions and sensory information between the two hemispheres. Anatomically, the CC has four distinct segments (rostrum, genu, body and splenium), all identifiable by 20 weeks post-conception (Edwards et al., 2014). In this study, we extended the identification of the cases with callosal hypoplasia by assessing the presence of these segments, and determining whether the hypoplasia was primarily related to thinning and / or shortening of the CC. We found no between-group differences in the distinct forms of callosal hypoplasia. However, we identified one case with partial agenesis of the CC (Fig. 1 panel E), characterized by markedly reduced length as a result of missing segments, primarily the splenium. This may result from an early perturbation of callosal development preceding the 20th week of gestation. The remaining cases of callosal hypoplasia are more likely related to insults later in gestation, leading to a reduction in size of the fully formed CC (De León Reyes et al., 2020). The development of the CC is regulated through a complex interplay of genes (see Edwards et al., 2014 for a comprehensive review), a number of which have been already linked to ASD (e.g., 17p13.3 which contains the *LIS1* gene, or 11q13.4 for the *DHCR7* gene; Edwards et al., 2014).

Additionally, we found a higher incidence of qualitative abnormalities of the opercular formation in ASD (Fig. 1 panel D_1-2_). This finding is unprecedented in ASD research, and is likely due to the fact that we assessed the opercular region explicitly, and objectified our findings by measuring inter-opercular distances. Our result converges with previous automated quantitative studies that repeatedly identified cortical morphometric changes predominantly in fronto-temporal and fronto-parietal regions in ASD (Hadjikhani et al., 2006, Hyde et al., 2010, Ecker et al., 2013). Recently, differences in fronto-temporal cortical thickness were also reported in a large sample derived from the same cohort as the present study (LEAP) (Ecker et al., 2022). Our finding also aligns with a previous report of cortical shape abnormalities specifically in the opercular region in ASD (Nordahl et al., 2007). Investigation of the link between qualitative anomalies of the opercular regions and morphometric changes in the pertaining cortical areas is a fascinating and open area of research.

The operculum is a large cortical structure encompassing parts of the frontal, temporal and parietal lobes, which together cover the insula. Functionally, the operculum is involved in social, sensory, language, and cognitive processing (Chen et al., 1996, Nordahl et al., 2007). Problems with these abilities are some of the core symptoms of ASD (American Psychiatric Association, 2013). The open operculum, resulting in an exposed insula, is not merely due to a volumetric reduction of frontal, temporal or parietal regions, but may be related to a disturbed developmental process, starting around 20–22 weeks’ gestation period and usually proceeding in a clear and well-orchestrated manner, known as opercularization. Therefore, our refined anatomical characterization of the opercular region may provide clues to the neurodevelopmental mechanisms involved.

In addition to micro- and macrocephaly, we found a few rare malformations of cortical development (MCD) in ASD, namely periventricular nodular heterotopia (PNH, n = 2, Fig. 1 panel B), diffuse simplified gyral pattern (n = 2, see Fig. 1 panel C), and cortical dysplasia (n = 3). Although there were no between-group differences due to the low incidence of these malformations, they are each suggestive of disrupted specific phases of cortical development, possibly related to specific genetic mutations. For example, PNH are disorders of the last phase of neuronal migration associated to, among others, *FLNA* or *ARFGEF2* mutations. Identification of genes associated with MCD (Barkovich et al., 2012, Desikan and Barkovich, 2016) in these participants may link between neuroradiological findings to neurodevelopment, and potentially to individual clinical profiles.

In sum, our study shows high prevalence of specific brain anomalies in ASD that may act as markers of neurodevelopmental processes involved. Furthermore, our results showed that neuroradiological findings were more likely to occur in isolation in controls, whereas they were more commonly associated with multiple findings in ASD. This converges with another recent study of cortical morphometry in the LEAP cohort, which suggested that the total amount of widespread deviation from typical brain anatomy is a better predictor of the clinical outcome in ASD than changes in any specific brain region per se (Pretzsch et al., 2022). Speculatively, these findings suggest a possible ‘cumulative effect’ of neurodevelopmental events in developing ASD.

Plausibly, some neuroradiological findings may cluster due to shared biological or mechanical factors (i.e., tissue viscoelasticity) which modulate the whole brain and shape of the neurocranium (Bilston, 2011). Our study confirms this hypothesis, as we indeed found that there were anomalies that tended to cluster together. For example, we found correlations between macrocephaly and ventriculomegaly, where progressive enlargement of intracranial ventricular system may have resulted in an abnormally large head (Orrù et al., 2018). We also found an association between macrocephaly and thickening of cranial bones, hinting at another possible mechanism for the increase in head size frequently observed in ASD (Lainhart et al., 1997, Fombonne et al., 1999, Dementieva et al., 2005, Orrù et al., 2018) in addition to early brain overgrowth (Courchesne, 2002).

Yet, we also found that neither the type, nor the number of neuroradiological findings per person, nor the pattern of association between different findings were specifically associated with the autism spectrum. In fact, there were no differences in neuroradiological findings between ASD and controls within the subsample of participants with mild ID, although this sample was relatively small. Previous studies including individuals with ID did not perform direct pairwise comparisons between diagnostic groups (Zeegers et al., 2006, Erbetta et al., 2015). Nevertheless, the literature on neuroradiological findings in ASD unanimously concurs that there are *no* specific individual (or association of) findings that are unique to ASD (Piven et al., 1990, Zeegers et al., 2006, Boddaert et al., 2009, Vasa et al., 2012, Erbetta et al., 2014, Erbetta et al., 2015, Monterrey et al., 2017, Myers et al., 2020). Our study corroborates this, further supporting the notion that brain imaging *per se* does not a have a direct role in the diagnosis of autism.

However, neuroradiological findings may be related to specific etiopathological mechanisms, which in turn, may be linked to ASD. Hence, our study illustrates that brain imaging has potential clinical relevance, particularly for evaluation of individual subjects (e.g. clinical genetics), and certainly in case of accompanying neurological and clinical signs and symptoms. From a methodological perspective, our study shows that detailed qualitative radiological screening of MRI scans is a valuable complement to automated (quantitative) assessments of brain morphometry.

Strengths of this study were its large sample size, and the use of a systematic and comprehensive scoring system of brain anomalies on MRI, augmented with standardized biometric measures of brain features. However, our findings must also be interpreted in the light of several limitations. First, T2w and FLAIR sequences were not available for all participants. However, these are not strictly necessary for identifying most of the MRI findings (e.g., persistent cavum septum pellucidum (Dremmen et al., 2019)). In addition, the acquisition parameters of the MRI scans used in this study were sub-optimal for assessing hippocampus. Therefore, analyses in this region must be interpreted with caution. Nonetheless, we ensured that all the reported findings in this study were adequately characterized by the available MRI sequences, and the number of scans missing did not differ between participants with and without ASD. Furthermore, not all scans in this study were reviewed independently by more than one rater. However, we worked in an interdisciplinary team, with one experienced, senior neuroradiologist supervising two pediatric neurologists with experience in brain imaging and development. Difficult scans were reviewed for consensus, and estimates of interrater agreement were remarkably high.

## 5 Conclusions

We used a systematic and comprehensive scoring system of brain anomalies on MRI, augmented with standardized biometric measures of brain features, and found a high incidence of neuroradiological findings in individuals with and without ASD. We found that neuroradiological findings were more common and clustered more frequently in ASD. Also, individual findings or clusters of findings may point towards the timing of neurodevelopmental mechanisms involved in individual cases. As such, clinical MRI assessments may be useful in the context of (genetic) diagnoses, and are potentially valuable to further elucidate the pathogenesis of autism.

## CRediT authorship contribution statement

**Sara Ambrosino:** Conceptualization, Methodology, Investigation, Data curation, Formal analysis, Writing – original draft, Writing – review & editing, Visualization. **Hasnaa Elbendary:** Methodology, Investigation. **Maarten Lequin:** Methodology, Investigation, Supervision, Writing – review & editing. **Dominique Rijkelijkhuizen:** Investigation. **Tobias Banaschewski:** Supervision, Writing – review & editing. **Simon Baron-Cohen:** Supervision. **Nico Bast:** Investigation, Writing – review & editing. **Sarah Baumeister:** Investigation, Writing – review & editing. **Jan Buitelaar:** Supervision, Writing – review & editing. **Tony Charman:** Supervision, Writing – review & editing. **Daisy Crawley:** Investigation. **Flavio Dell'Acqua:** Data curation. **Hannah Hayward:** Investigation. **Rosemary Holt:** Investigation, Writing – review & editing. **Carolin Moessnang:** Investigation, Writing – review & editing. **Antonio M. Persico:** Supervision, Writing – review & editing. **Roberto Sacco:** Investigation, Writing – review & editing. **Antonia San José Cáceres:** Investigation, Writing – review & editing. **Julian Tillmann:** Data curation, Writing – review & editing. **Eva Loth:** Supervision, Funding acquisition. **Christine Ecker:** Writing – review & editing. **Bob Oranje:** Writing – review & editing, Supervision. **Declan Murphy:** Writing – review & editing, Supervision, Funding acquisition. **Sarah Durston:** Writing – review & editing, Supervision.

## Declaration of Competing Interest

The authors declare the following financial interests/personal relationships which may be considered as potential competing interests: TB served in an advisory or consultancy role for ADHS digital, Infectopharm, Lundbeck, Medice, Neurim Pharmaceuticals, Oberberg GmbH, Roche, and Takeda. He received conference support or speaker’s fee by Medice and Takeda. He received royalities from Hogrefe, Kohlhammer, CIP Medien, Oxford University Press; the present work is unrelated to these relationships. JKB has been in the past 3 years a consultant to / member of advisory board of / and/or speaker for Takeda/Shire, Roche, Medice, Angelini, Janssen, and Servier. He is not an employee of any of these companies, and not a stock shareholder of any of these companies. He has no other financial or material support, including expert testimony, patents, royalties. ASJC has worked with Servier and been a member of advisory boards for Servier and Roche. JT is an employee of F. Hoffmann–La Roche ltd. The other authors report no biomedical financial interests or potential conflicts of interest.
