# Supplementary material for: In-depth characterization of neuroradiological findings in a large sample of individuals with autism spectrum disorder and controls
Source: Neuroimage Clin. 2022 Jul 16;35:103118. doi: 10.1016/j.nicl.2022.103118 (PMC9421485; doi:10.1016/j.nicl.2022.103118)
Supplement: Appendix A [file mmc1.docx]

**Supporting Online Information for ‘In-depth characterization of neuroradiological findings in a large sample of individuals with autism spectrum disorder and controls’ by Ambrosino et al.**

**Appendix A: Scanning parameters**

| Site | Manufacturer | Model | Software version | Acquisition sequence | Slices  (n) | TR  (s) | TE  (ms) | FA  (˚) | Coverage | | Voxel size (mm^3^) | FOV  (mm) |
| --- | --- | --- | --- | --- | --- | --- | --- | --- | --- | --- | --- | --- |
|  |  |  |  |  |  |  |  |  |  | |  |  |
|  |  |  |  |  |  |  |  |  |  | |  |  |
| Cambridge | Siemens | Verio | Syngo MR B17 | Tfl3d1_ns | 176 | 2.30 | 2.95 | 9 | 256*256 | 1.1*1.1*1.2 | | 270 |
|  |  |  |  | Ax FLAIR | 27 | 7.84 | 96 | 150 | 240*320 | 0.7*0.7*4 | | 224 |
|  |  |  |  |  |  |  |  |  |  |  | |  |
|  |  |  |  |  |  |  |  |  |  |  | |  |
| KCL | GE  Medical systems | Discovery mr750 | LX MR DV23.1_V02_1317.c | SAG ADNI GO ACC SPGR | 196 | 7.31 | 3.02 | 11 | 256*256 | 1.1*1.1*1.2 | | 270 |
|  |  |  |  | Ax T2 FRFSE | 72 | 4.38 | 60 | / | 512*512 | 0.5*0.5*2 | | 240 |
|  |  |  |  |  |  |  |  |  |  |  | |  |
|  |  |  |  | Ax T2 FLAIR | 36 | 8.00 | 120 | / | 512*512 | 0.4*0.4*4 | | 220 |
|  |  |  |  |  |  |  |  |  |  |  | |  |
|  |  |  |  |  |  |  |  |  |  |  | |  |
| Mannheim | Siemens | TimTrio | Syngo MR B17 | MPRAGE ADNI | 176 | 2.30 | 2.93 | 9 | 256*256 | 1.1*1.1*1.2 | | 270 |
|  |  |  |  |  |  |  |  |  |  |  | |  |
| Nijmegen | Siemens | Skyra | Syngo MR D13 | Tfl3d1_16ns | 176 | 2.30 | 2.93 | 9 | 256*256 | 1.1*1.1*1.2 | | 270 |
|  |  |  |  | T2 | 25 | 1.50 | 80 | 150 | 320*320 | 0.7*0.7*4 | | 220 |
|  |  |  |  |  |  |  |  |  |  |  | |  |
|  |  |  |  |  |  |  |  |  |  |  | |  |
| Rome | GE  Medical systems | Signa HDxt | 24/LX/MR HD16.0_V02_1131.a | SAG ADNI GO ACC SPGR | 172 | 5.96 | 1.76 | 11 | 256*256 | 1.1*1.1*1.2 | | 270 |
|  |  |  |  | ACPC T2 | 72 | 5.78 | 127 | 90 | 512*512 | 0.5*0.5*2 | | 240 |
|  |  |  |  |  |  |  |  |  |  |  | |  |
|  |  |  |  | Ax T2 FLAIR | 36 | 9.00 | 122 | 90 | 512*512 | 0.5*0.5*4 | | 240 |
|  |  |  |  |  |  |  |  |  |  |  | |  |
|  |  |  |  |  |  |  |  |  |  |  | |  |
| Utrecht | Philips  Medical Systems | Achieva | 3.2.3/3.2.3.1 | ADNI GO 2 | 170 | 6.76 | 3.1 | 9 | 256*256 | 1.1*1.1*1.2 | | 270 |
|  |  |  |  |  |  |  |  |  |  |  | |  |

**Table A1**. Scanner details and acquisition parameters at each participating site

Abbreviations: n, number; TR, repetition time; TE, echo time; FA, flip angle; FOV, field of view
