# Supplementary material for: In-depth characterization of neuroradiological findings in a large sample of individuals with autism spectrum disorder and controls
Source: Neuroimage Clin. 2022 Jul 16;35:103118. doi: 10.1016/j.nicl.2022.103118 (PMC9421485; doi:10.1016/j.nicl.2022.103118)
Supplement: Appendix C [file mmc3.docx]

**Supporting Online Information for ‘In-depth characterization of neuroradiological findings in a large sample of individuals with autism spectrum disorder and controls’ by Ambrosino et al.**

**Appendix C: Biometric assessments**

**Head size and proportions**

To assess head size and shape, we measured the greatest Anterior-posterior (AP) diameter from the T1 mid-sagittal plane, and the greatest Bi-parietal (BP) diameter from the T1 axial plane that most closely aligned with the AP diameter. Both diameters usually went through or were close to the thalami.

The Cranial Index (CI) was calculated as the ratio of head-width (BP) expressed as a percentage of head-length (AP). Accordingly, CI was computed as: CI = (BP / AP) * 100.

Head circumference was computed: HC= (BP + AP) * Π / 2.

Extreme deviations from the three main head shapes (brachycephalic, mesocephalic, and dolichocephalic types) were estimated. Hyperdolichocephaly was defined as CI < 71, hyperbrachycephaly was defined as CI > 90 (Franco et al., 2013). Plagiocephaly was confirmed by measuring Cranial Vault Asymmetry (CVA) > 5 mm.

Macro- and microcephaly were defined in case of HC > 97^th^ or < 3^rd^ percentiles, respectively (Allanson et al., 2009).

**Inter-opercular distances**

Underdevelopment of the opercular region (an “open” operculum) was defined as an increased mean distance (> 3.5 mm) between the posterior-inferior border of the inferior frontal gyrus and the superior-anterior border of the temporal lobe (anterior open operculum) on sagittal (*a*) and axial (*b*) planes, or an increased mean distance (> 0.5 mm) between the inferior border of the parietal operculum and the superior border of the temporal operculum (posterior open operculum) in sagittal (*c*) or coronal (*d*) planes. Mean anterior and posterior interopercular distances of each hemisphere were calculated by averaging *a* and *b*, or *c* and *d*, respectively. (Chen et al. 1995, Chen et al. 1996).

The **Corpus callosum** is the largest commissural tract, structurally divided into four segments: rostrum, genu, body and splenium. We assessed the anterior-posterior diameter of the corpus callosum by measuring the distance between the anterior aspect of the genu and the posterior aspect of the splenium on midline sagittal sections. The thickness of the corpus callosum was measured at the level of the body. Callosal measures that were less than the 3^rd^ percentile or greater than the 97^th^ percentile (Garel et al. 2011, Karakaş et al. 2011) were considered to be short/thin or long/thick corpus callosum, respectively.

On the midsagittal plane we also measured the largest diameter of **pineal gland** cysts, if present.

In the posterior fossa, **mega cisterna magna** was defined as a $\geq$10 mm enlargement of the retro and infra-cerebellar cerebrospinal fluid (CSF) space measured between the inferior margin of the vermis and the posterior rim of the foramen magnum perpendicular to the occipital dura on the midsagittal plane, in the presence of an intact vermis and a normal 4^th^ ventricle (Limperopoulos et al., 2009).

**Chiari** **malformation type 1** was defined in case of herniation of cerebellar tonsils $\geq$ 5 mm below the foramen magnum (Baisden, 2012).

**Ventriculomegaly**

Evans' index was measured on transverse images as the ratio between the maximum diameter of the frontal horns of the lateral ventricles and the maximum inner diameter of the skull in the same section. Ventriculomegaly was graded as follows: 0, normal (Evans' index < 0.3); 1, slight dilation (Evans' index 0.3–0.35); or 2, dilation (Evans' index > 0.35) (Sari et al., 2015).

The septum pellucidum is a vertical double membrane separating the anterior horns of the lateral ventricles. The two layers are separated at birth and typically fuse into a single septum within 5 months, likely due to the growth of the surrounding brain structures (Sarwar, 1989). Occasionally, this process fails leaving a persistent cavum. A cavum length 1-4 mm is common in a large proportion of healthy subjects. In accordance to most previous studies, we defined an enlarged **cavum septum pellucidum** in case of a cavum length of ≥ 6 mm (Dremmen et al., 2019).

Measurements of the subarachnoid space width were taken on coronal images, in cuts passing through the frontal horns of the lateral ventricles, as the maximum width of the CSF from the crest of a gyrus to the nearest point on the inner table of the skull. Subarachnoid CSF space enlargement was defined in case of a cranio-cortical width ≥ 6 mm (Marino et al., 2014)

Finally, we measured the size (largest diameter) of perivascular **Virchow-Robin (VR) spaces** in subcortical and deep white matter, and associated with the lenticulostriate arteries. According to the reference work of Heier and colleagues (1989), VR spaces were considered dilated if they were larger than 3 mm.

**References**

- Allanson, J.E., Cunniff, C., Hoyme, H.E., McGaughran, J., Muenke, M., Neri, G., 2009. Elements of morphology: standard terminology for the head and face. Am. J. Med. Genet. A. Jan;149A(1), 6-28. doi: 10.1002/ajmg.a.32612. PMID: 19125436; PMCID: PMC2778021.
- Baisden, J., 2012. Controversies in Chiari I malformations. Surg. Neurol. Int. 3(Suppl 3), S232–S237. https://doi.org/10.4103/2152-7806.98580
- Chen, C.Y., Zimmerman, R.A., Faro, S., Parrish, B., Wang, Z., Bilaniuk, L.T., Chou, T.Y., 1995. MR of the cerebral operculum: topographic identification and measurement of interopercular distances in healthy infants and children. AJNR Am. J. Neuroradiol. Sep;16(8), 1677-87. PMID: 7502974.
- Chen, C.Y., Zimmerman, R.A., Faro, S., Parrish, B., Wang, Z., Bilaniuk, L.T., Chou T.Y., 1996. MR of the cerebral operculum: abnormal opercular formation in infants and children. AJNR Am. J. Neuroradiol. Aug;17(7), 1303-11. PMID: 8871716.
- Dremmen, M.H.G., Bouhuis, R.H., Blanken, L.M.E., Muetzel, R.L., Vernooij, M.W., Marroun, H.E., Jaddoe, V.W.V., Verhulst, F.C., Tiemeier, H., White T., 2019. Cavum Septum Pellucidum in the General Pediatric Population and Its Relation to Surrounding Brain Structure Volumes, Cognitive Function, and Emotional or Behavioral Problems. AJNR Am. J. Neuroradiol. Feb;40(2), 340-346. doi: 10.3174/ajnr.A5939. PMID: 30679220; PMCID: PMC7028615.
- Franco, F.C., de Araujo, T.M., Vogel, C.J., Quintão, C.C., 2013. Brachycephalic, dolichocephalic and mesocephalic: Is it appropriate to describe the face using skull patterns? Dental Press J. Orthod. May-Jun;18(3):159-63. doi: 10.1590/s2176-94512013000300025. PMID: 24094027.
- Garel, C., Cont, I., Alberti, C., Josserand, E., Moutard, M.L., Ducou le Pointe, H., 2011. Biometry of the corpus callosum in children: MR imaging reference data. AJNR Am. J. Neuroradiol. Sep;32(8), 1436-43. doi: 10.3174/ajnr.A2542. Epub 2011 Jul 28. PMID: 21799035; PMCID: PMC7964359.
- Heier, L.A., Bauer, C.J., Schwartz, L., Zimmerman, R.D., Morgello, S., Deck M.D., 1989. Large Virchow-Robin spaces: MR-clinical correlation. AJNR Am. J. Neuroradiol. Sep-Oct;10(5), 929-36. PMID: 2505536.
- Karakaş, P., Koç, Z., Koç, F., Gülhal Bozkır, M., 2011. Morphometric MRI evaluation of corpus callosum and ventricles in normal adults. Neurol. Res. Dec;33(10), 1044-9. doi: 10.1179/1743132811Y.0000000030. PMID: 22196757.
- Limperopoulos, C., Robertson, R. L., Jr Khwaja, O. S., Robson, C. D., Estroff, J. A., Barnewolt, C., Levine, D., Morash, D., Nemes, L., Zaccagnini, L., du Plessis, A. J., 2008. How accurately does current fetal imaging identify posterior fossa anomalies?. AJR. Am. J. Roentgenol. 190(6), 1637–1643. <https://doi.org/10.2214/AJR.07.3036>
- Marino, M.A., Morabito R., Vinci, S., Germanò, A., Briguglio, M., Alafaci, C., Mormina, E., Longo, M., & Granata F., 2014. Benign external hydrocephalus in infants. A single centre experience and literature review. Neuroradiol. J. 27(2), 245–250. https://doi.org/10.15274/NRJ-2014-10020
- Sarı, E., Sarı, S., Akgün, V., Özcan, E., Ìnce, S., Babacan, O., Saldır, M., Açıkel, C., Başbozkurt, G., Yeşilkaya, Ş., Kılıc, C., Kara, K., Vurucu, S., Kocaoğlu, M., Yeşilkaya, E., 2015. Measures of ventricles and evans' index: from neonate to adolescent. Pediatr. Neurosurg. 50(1), 12-7. doi: 10.1159/000370033. Epub 2015 Jan 22. PMID: 25613691.
- Sarwar, M., 1989. The septum pellucidum: normal and abnormal. AJNR Am. J. Neuroradiol. Sep-Oct;10(5), 989-1005. PMID: 2505543; PMCID: PMC8335275.
