# Supplementary material for: In-depth characterization of neuroradiological findings in a large sample of individuals with autism spectrum disorder and controls
Source: Neuroimage Clin. 2022 Jul 16;35:103118. doi: 10.1016/j.nicl.2022.103118 (PMC9421485; doi:10.1016/j.nicl.2022.103118)
Supplement: Appendix D [file mmc4.docx]

**Supporting Online Information for ‘In-depth characterization of neuroradiological findings in a large sample of individuals with autism spectrum disorder and controls’ by Ambrosino et al.**

**Appendix D: Supplementary results**

|  | Total  N=620 | Male  n=428 | | Female  n=192 | | Sex diff*  *p* value | | |  |  |
| --- | --- | --- | --- | --- | --- | --- | --- | --- | --- | --- |
| Head, brain, and lobes |  |  | |  | |  | | |  |  |
|  |  |  | |  | |  | | |  |  |
| Cranial deformity All | 28 (4.5%) | 18 (4.2%) | | 10 (5.2%) | | ns | | |  |  |
| Plagiocephaly | 9 (1.5%) | 8 (1.9%) | | 1 (0.5%) | | ns | | |  |  |
| Hyperbrachycephaly | 9 (1.5%) | 6 (1.4%) | | 3 (1.6%) | | ns | | |  |  |
| Hyperdolichocephaly | 10 (1.6%) | 6 (1.4%) | | 4 (2.1%) | | ns | | |  |  |
|  |  |  | |  | |  | | |  |  |
| Cranial volume All | 133 (21.5%) | 86 (20.1%) | | 47 (24.5%) | | ns | | |  |  |
| Microcephaly | 47 (7.6%) | 7 (1.6%) | | 40 (20.8%) | | **< .001** | | |  |  |
| Macrocephaly | 86 (13.9%) | 79 (18.5%) | | 7 (3.6%) | | **< .001** | | |  |  |
|  |  |  | |  | |  | | |  |  |
| Calvarian / dural thickening | 126 (20.3%) | 95 (22.2%) | | 31 (16.1%) | | ns | | |  |  |
|  |  |  | |  | |  | | |  |  |
| Opercular abnormalities | 229 (36.9%) | 160 (37.4%) | | 69 (35.9%) | | ns | | |  |  |
|  |  |  | |  | |  | | |  |  |
| Cerebral cortex |  |  | |  | |  | | |  |  |
|  |  |  | |  | |  | | |  |  |
| Malformations All | 8 (1.3%) | 7 (1.6%) | | 1 (0.5%) | | ns | | |  |  |
| Periventricular nodular Heterotopia | 3 (0.5%) | 3 (0.7%) | | 0 (0%) | | ns | | |  |  |
| Simplified gyral pattern | 2 (0.3%) | 2 (0.5%) | | 0 (0%) | | ns | | |  |  |
| Cortical dysplasia | 3 (0.5%) | 2 (0.5%) | | 1 (0.5%) | | ns | | |  |  |
|  |  |  | |  | |  | | |  |  |
| Lesions | 3 (0.5%) | 3 (0.7%) | | 0 (0%) | | ns | | |  |  |
|  |  |  | |  | |  | | |  |  |
| Hippocampi |  |  | |  | |  | | |  |  |
|  |  |  | |  | |  | | |  |  |
| Lesions | 2 (0.3%) | 1 (0.2%) | | 1 (0.5%) | | ns | | |  |  |
|  |  |  | |  | |  | | |  |  |
| White matter |  |  | |  | |  | | |  |  |
|  |  |  | | |  | | |  | | |
| Lesions | 15 (2.4%) | 12 (2.8%) | 3 (1.6%) | | | | ns | |  |  |
|  |  |  |  | | | |  | |  |  |
| Virchow-Robin spaces |  |  |  | | | |  | |  |  |
|  |  |  |  | | | | |  | |  |
| Dilation All | 338 (54.5%) | 243 (56.8%) | 95 (49.5%) | | | | | ns | |  |
| Deep white matter / subcortical | 133 (21.5%) | 104 (24.3%) | 29 (15.1%) | | | | | .010 | |  |
| Lenticulo-striate | 300 (48.4%) | 216 (50.5%) | 84 (43.8%) | | | | | ns | |  |
|  |  |  |  | | | | |  | |  |
| Basal ganglia | 0 (0%) | 0 (0%) | 0 (0%) | | | | | ns | |  |
|  |  |  |  | | | | |  | |  |

| Posterior fossa |  |  |  |  |
| --- | --- | --- | --- | --- |
| All | 115 (18.5%) | 96 (22.4%) | 19 (9.9%) | **< .001** |
|  |  |  |  |  |
| Dandy-Walker complex | 106 (17.1%) | 92 (21.5%) | 14 (7.3%) | **< .001** |
| Mega cisterna magna | 82 (13.2%) | 73 (17.1%) | 9 (4.7%) | **< .001** |
| Dandy-Walker variant | 3 (0.5%) | 3 (0.7%) | 0 (0%) | ns |
| Blake pouch cyst | 1 (0.2%) | 1 (0.2%) | 0 (0%) | ns |
| Arachnoid cyst | 13 (2.1%) | 12 (2.8%) | 1 (0.5%) | ns |
| Vermian hypoplasia | 7 (1%) | 3 (0.7%) | 4 (2.1%) | ns |
|  |  |  |  |  |
| Chiari type 1 malformation | 7 (1.1%) | 3 (0.7%) | 4 (2.1%) | ns |
|  |  |  |  |  |
| Lesion | 1 (0.2%) | 1 (0.2%) | 0 (0%) | ns |
|  |  |  |  |  |
| Vascular anomaly | 1 (0.2%) | 0 (0%) | 1 (0.5%) | ns |
|  |  |  |  |  |
| CSF spaces |  |  |  |  |
|  |  |  |  |  |
| Ventriculomegaly | 26 (4.2%) | 21 (4.9%) | 5 (2.6%) | ns |
|  |  |  |  |  |
| Cavum septum pellucidum / vergae | 12 (1.9%) | 9 (2.1%) | 3 (1.6%) | ns |
|  |  |  |  |  |
| Choroid plexus cysts | 3 (0.5%) | 3 (0.7%) | 0 (0%) | ns |
|  |  |  |  |  |
| Subarachnoid spaces Enlargement | 57 (9.2%) | 19 (4.4%) | 38 (19.8%) | ns |
|  |  |  |  |  |
| Calcifications | 2 (0.3%) | 2 (0.5%) | 0 (0%) | ns |
|  |  |  |  |  |
| Midline |  |  |  |  |
|  |  |  |  |  |
| CC Hypoplasia All | 30 (4.8%) | 16 (3.7%) | 14 (7.3%) | ns |
| CC thin | 10 (1.6%) | 3 (0.7%) | 7 (3.6%) | .012 |
| CC short | 12 (1.9%) | 8 (1.9%) | 4 (2.1%) | ns |
| CC short and thin | 4 (0.6%) | 3 (0.7%) | 1 (0.5%) | ns |
| CC partial agenesis | 1 (0.2%) | 1 (0.2%) | 0 (0%) | ns |
| CC focal hypoplasia | 4 (0.6%) | 2 (0.5%) | 2 (1.0%) | ns |
|  |  |  |  |  |
| Pineal gland cyst All | 90 (14.5%) | 58 (13.6%) | 32 (16.7%) | ns |
| ≥ 10 mm | 14 (2.3%) | 9 (2.1%) | 5 (2.6%) | ns |
| < 10 mm | 76 (12.3%) | 49 (11.4%) | 27 (14.1%) | ns |
|  |  |  |  |  |
| Other |  |  |  |  |
| Vascular anomalies All | 10 (1.6%) | 4 (0.9%) | 6 (3.1%) | ns |
| DVA | 8 (1.3%) | 3 (0.7%) | 5 (2.6%) | ns |
| Kissing carotids | 1 (0.2%) | 0 (0%) | 1 (0.5%) | ns |
| Capillary teleangectasia | 1 (0.2%) | 1 (0.2%) | 0 (0%) | ns |
|  |  |  |  |  |
| Cysts All | 8 (1.3%) | 6 (1.4%) | 2 (1.0%) | ns |
| Arachnoid** | 3 (0.5%) | 3 (0.7%) | 0 (0%) | ns |
| Poroencefalic | 3 (0.5%) | 2 (0.5%) | 1 (0.5%) | ns |
| Neuroglial | 1 (0.2%) | 1 (0.2%) | 0 (0%) | ns |
| Inclusion | 1 (0.2%) | 0 (0%) | 1 (0.5%) | ns |

**Table D.1**. Neuroradiological findings – sex differences

Abbreviations: ASD, autism spectrum disorder; n, number; M, mean; SD, standard deviation; ns, not significant; IQ, intelligence quotient; ID, intellectual disability; CSF, cerebral spinal fluid; CC, corpus callosum; DVA, developmental venous anomalies. Note: *Χ^2^ or Fisher’s Exact Test, as appropriate, for testing for sex differences (raw p-values; results reaching significance when controlling FDR are indicated in bold). **Arachnoid cysts in locations other than the posterior fossa.

|  | **1** | **2** | **3** | **4** | **5** | **6** | **7** | **8** | **9** | **10** | **11** | **12** | **13** | **14** | **15** | **16** | **17** | **18** | **19** | **20** | **21** | **22** | **23** | **24** | **25** |
| --- | --- | --- | --- | --- | --- | --- | --- | --- | --- | --- | --- | --- | --- | --- | --- | --- | --- | --- | --- | --- | --- | --- | --- | --- | --- |
| **1. Cranial deformity** | - | ns | ns | ns | ns | ns | ns | ns | ns | ns | ns | ns | ns | .036 | ns | ns | ns | ns | ns | ns | ns | ns | ns | ns | ns |
| **2. Microcephaly** | ns | - | - | ns | ns | ns | ns | ns | ns | ns | ns | ns | ns | ns | ns | ns | ns | ns | ns | ns | ns | **< .0001** | ns | ns | ns |
| **3. Macrocephaly** | ns | - | - | **< .0001** | **.**047 | ns | ns | ns | .017 | ns | ns | ns | ns | ns | ns | ns | **< .0001** | ns | ns | ns | ns | ns | ns | ns | ns |
| **4. Thickening dura / skull** | ns | ns | **< .0001** | - | **< .0001** | ns | ns | ns | ns | ns | ns | ns | ns | .034 | .034 | ns | ns | ns | ns | .027 | ns | ns | ns | ns | ns |
| **5. Opercula abnormality** | ns | ns | .047 | **< .0001** | - | ns | ns | ns | ns | ns | ns | ns | ns | ns | ns | ns | .002 | ns | ns | .004 | ns | .002 | ns | ns | ns |
| **6. Cortical malformation** | ns | ns | ns | ns | ns | - | ns | **< .0001** | ns | ns | ns | ns | ns | ns | ns | ns | ns | ns | ns | ns | ns | ns | ns | ns | **< .0001** |
| **7. Hippocampi** | ns | ns | ns | ns | ns | ns | - | ns | ns | ns | ns | ns | ns | ns | ns | ns | ns | .038 | ns | ns | ns | ns | ns | ns | .026 |
| **8. White Matter lesions** | ns | ns | ns | ns | ns | **< .0001** | ns | - | ns | ns | ns | ns | ns | ns | .011 | ns | ns | ns | ns | ns | ns | ns | ns | ns | .001 |
| **9. Virchow-Robin spaces** | ns | ns | ns | ns | ns | ns | ns | ns | - | ns | ns | ns | ns | ns | ns | ns | ns | ns | ns | ns | ns | ns | ns | ns | ns |
| **10. Mega cisterna magna** | ns | ns | ns | ns | ns | ns | ns | ns | ns | - | - | - | - | - | ns | ns | ns | ns | ns | .025 | .017 | ns | ns | ns | ns |
| **11. Dandy-Walker variant** | ns | ns | ns | ns | ns | ns | ns | ns | ns | - | - | - | - | - | ns | ns | .005 | ns | ns | .023 | ns | ns | ns | ns | ns |
| **12. Blake cyst** | ns | ns | ns | ns | ns | ns | ns | ns | ns | - |  | - | - | - | ns | ns | .042 | ns | ns | ns | ns | .048 | ns | ns | ns |
| **13. Cerebellar cysts** | ns | ns | ns | ns | ns | ns | ns | ns | ns | - | - | - | - | - | ns | ns | .014 | ns | ns | ns | ns | ns | ns | ns | ns |
| **14. Vermis hypoplasia** | .036 | ns | ns | .034 | ns | ns | ns | ns | ns | - | - | - | - | - | ns | ns | ns | ns | ns | ns | ns | .041 | ns | ns | ns |
| **15. Chiari 1 malformation** | ns | ns | ns | .034 | ns | ns | ns | .011 | ns | ns | ns | ns | ns | ns | - | ns | ns | ns | ns | ns | ns | ns | ns | ns | ns |
| **16. Cerebellar lesions** | ns | ns | ns | ns | ns | ns | ns | ns | ns | ns | ns | ns | ns | ns | ns | - | ns | ns | ns | ns | ns | ns | ns | .032 | ns |
| **17. Ventriculomegaly** | ns | ns | **< .0001** | ns | .002 | ns | ns | ns | ns | ns | .005 | .042 | .013 | ns | ns | ns | - | ns | ns | .001 | ns | ns | ns | ns | ns |
| **18. Cavum septum** | ns | ns | ns | ns | ns | ns | .038 | ns | ns | ns | ns | ns | ns | ns | ns | ns | ns | - | ns | ns | ns | ns | ns | ns | ns |
| **19. Choroid cysts** | ns | ns | ns | ns | ns | ns | ns | ns | ns | ns | ns | ns | ns | ns | ns | ns | ns | ns | - | ns | ns | .007 | ns | ns | ns |
| **20. Periencephalic spaces** | ns | ns | ns | .027 | .004 | ns | ns | ns | ns | .025 | .023 | ns | ns | ns | ns | ns | .001 | ns | ns | - | ns | ns | ns | ns | ns |
| **21. Calcifications** | ns | ns | ns | ns | ns | ns | ns | ns | ns | .017 | ns | ns | ns | ns | ns | ns | ns | ns | ns | ns | - | ns | ns | ns | ns |
| **22. CC hypoplasia** | ns | **< .0001** | ns | ns | .002 | ns | ns | ns | ns | ns | ns | .048 | ns | .041 | ns | ns | ns | ns | .007 | ns | ns | - | ns | ns | ns |
| **23. Pineal gland cyst** | ns | ns | ns | ns | ns | ns | ns | ns | ns | ns | ns | ns | ns | ns | ns | ns | ns | ns | ns | ns | ns | ns | - | ns | ns |
| **24. Vascular abnormality** | ns | ns | ns | ns | ns | ns | ns | ns | ns | ns | ns | ns | ns | ns | ns | .032 | ns | ns | ns | ns | ns | ns | ns | - | ns |
| **25. Other Cysts** | ns | ns | ns | ns | ns | **< .0001** | .026 | .001 | ns | ns | ns | ns | ns | ns | ns | ns | ns | ns | ns | ns | ns | ns | ns | ns | - |

**Table D.2**. Correlations between neuroradiological findings (p-values).

Note. Correlations reaching Bonferroni-corrected significance are indicated in bold.
